# Mapping a male-fertility restoration locus for the A_4_ cytoplasmic-genic male-sterility system in pearl millet using a genotyping-by-sequencing-based linkage map

**DOI:** 10.1186/s12870-018-1267-8

**Published:** 2018-04-17

**Authors:** Anna Pucher, C. Tom Hash, Jason G. Wallace, Sen Han, Willmar L. Leiser, Bettina I. G. Haussmann

**Affiliations:** 1 Institute of Plant Breeding, Seed Science and Population Genetics, Fruwirthstr University of Hohenheim, 21, D-70599 Stuttgart, Germany; 2 ICRISAT Sahelian Center, 12404 Niamey, BP Niger; 3 Department of Crop and Soil Sciences, the University of Georgia, 30602 Athens, GA USA; 4 State Plant Breeding Institute, University of Hohenheim, Fruwirthstr, 21, D-70599 Stuttgart, Germany

**Keywords:** Pearl millet, Male fertility restoration, Cytoplasmic male sterility, QTL mapping, Genotyping-by-sequencing

## Abstract

**Background:**

Pearl millet (*Pennisetum glaucum* (L.) R. Br., syn. *Cenchrus americanus* (L.) R. Br) is an important cereal and fodder crop in hot and arid environments. There is great potential to improve pearl millet production through hybrid breeding. Cytoplasmic male sterility (CMS) and the corresponding nuclear fertility restoration / sterility maintenance genes (*Rf*s) are essential tools for economic hybrid seed production in pearl millet. Mapping the *Rf* genes of the A_4_ CMS system in pearl millet would enable more efficient introgression of both dominant male-fertility restoration alleles (*Rf*) and their recessive male-sterility maintenance counterparts (*rf*).

**Results:**

A high density linkage map based on single nucleotide polymorphism (SNP) markers was generated using an F_2_ mapping population and genotyping-by-sequencing (GBS). The parents of this cross were ‘ICMA 02777’ and ‘ICMR 08888’, which segregate for the A_4_ *Rf* locus. The linkage map consists of 460 SNP markers distributed mostly evenly and has a total length of 462 cM. The segregation ratio of male-fertile and male-sterile plants (3:1) based on pollen production (presence/absence) indicated monogenic dominant inheritance of male-fertility restoration. Correspondingly, a major quantitative trait locus (QTL) for pollen production was found on linkage group 2, with cross-validation showing a very high QTL occurrence (97%). The major QTL was confirmed using selfed seed set as phenotypic trait, though with a lower precision. However, these QTL explained only 14.5% and 9.9% of the phenotypic variance of pollen production and selfed seed set, respectively, which was below expectation. Two functional KASP markers were developed for the identified locus.

**Conclusion:**

This study identified a major QTL for male-fertility restoration using a GBS-based linkage map and developed KASP markers which support high-throughput screening of the haploblock. This is a first step toward marker-assisted selection of A_4_ male-fertility restoration and male-sterility maintenance in pearl millet.

**Electronic supplementary material:**

The online version of this article (10.1186/s12870-018-1267-8) contains supplementary material, which is available to authorized users.

## Background

Pearl millet (*Pennisetum glaucum* (L.) R. Br., syn. *Cenchrus americanus* (L.) R. Br), a highly nutritious, drought- and salinity-tolerant cereal crop, is grown predominantly by subsistence farmers in semi-arid regions of West Africa and South Asia, where its yield levels are generally low due to limited water availability, high temperatures, and low soil fertility. Pearl millet is a naturally outcrossing species and benefits greatly from exploitation of heterosis [1]; hybrid breeding programs for this crop are already well established in India and are in the early stages of development in West Africa.

Cytoplasmic male sterility (CMS) is characterized by anthers failing to produce functional pollen while stigma develops normally. CMS occurs when recessively inherited nuclear genes interact with a male-sterility-inducing cytoplasm. CMS is thus maternally inherited and facilitates large-scale hybrid seed production by preventing self-pollination. CMS systems are utilized in pearl millet and many other hybrid crops for which grain or fruit is an economically important component of the harvest [2, 3]. Male-fertility can be restored in the background of the male-sterility-inducing cytoplasm by dominantly inherited nuclear restorer genes, termed *Rf* genes. These genes counteract the effects of the sterility-inducing genes in the cytoplasm (meaning mitochondria and/or chloroplasts) and allow the production of male-fertile hybrid plants [4].

In pearl millet, the first reported CMS system (A_1_) was based on the Tift 23A_1_ cytoplasm [5]. Subsequently, the A_2_, A_3_ & A_β_ systems were found as alternatives [6, 7]; however, these systems all proved to be less stable than the A_1_ CMS system, so the A_1_ system alone was used in hybrid pearl millet breeding in India for several decades. To avoid cytoplasmic uniformity, which can cause the vulnerability to disease and insect pest epidemics [8], alternative CMS sources to the A_1_ system were sought for cytoplasmic diversification in hybrid pearl millet. Several sources were studied [6, 9–13], but only the A_m_ = A_4_ and A_5_ CMS systems were identified as commercially viable [14, 15]. Other CMS systems did not satisfy the required attributes like complete male sterility of A-lines, high degree of male-fertility restoration of their hybrids and the stability of these traits across environments.

In West Africa, current activities include identifying promising hybrid parents, determining appropriate CMS system(s), and introgression of appropriate male-sterility maintenance/male-fertility restoration alleles into locally-adapted germplasm. A_4_ and A_5_ CMS systems appear to offer more stable male-sterility than A_1_ in the hotter production environments of West Africa, which agrees with higher rates of pollen shed in supposedly male-sterile plants under higher temperature conditions in India. Currently, the A_4_ and A_5_ *Rf* genes are most readily available in germplasm adapted to Indian conditions [16], and the frequency of maintainer alleles for both of these systems in West African pearl millet germplasm appears to be high [17]. Genetic mapping of *Rf* loci for the A_4_ and A_5_ CMS systems would enable more efficient transfer of these fertility restoration alleles into one or more potential male heterotic pools adapted to West African conditions. In addition, such mapping could facilitate further diversification of potential hybrid seed parents by making it easier to track and manipulate male-sterility genes as diverse germplasm is integrated into breeding programs.

Over the past three decades many different types of markers were developed and used for genetic mapping and/or diversity assessment in pearl millet, including restriction fragment length polymorphism markers (RFLPs), amplified fragment length polymorphism markers (AFLPs), simple sequence repeat markers (SSRs), diversity arrays technology markers (DArT™s), and single-nucleotide polymorphisms (SNPs). The quality of genetic maps improved by increasing marker density and coverage, but many maps based on RFLPs, AFLPs, and SSRs are still not satisfactory due to marker clustering in peri-centromeric regions and extremely high rates of recombination in peri-telomeric regions, which causes gaps greater than 20 cM [18–21].

SNP markers, which are abundant throughout the genome, are now commonly used in many crops. The low costs of high-throughput sequencing methods facilitate the development of high-density linkage maps based on SNP markers. Genotyping-by-sequencing (GBS) is one sequencing technique that is able to generate such genome-wide SNP datasets [22]. In the first step of the GBS method, genome complexity is reduced using restriction enzymes, which cut the genomic DNA selectively. In the next step, ‘barcoded’ DNA adapters are ligated to each fragment to enable sequencing of many samples in one sequencing lane. GBS has already proven its success in several crops like maize, barley, sorghum, and grapes. [22, 23]. Moumouni et al. [24] and Punnuri et al. [25] have shown that GBS can develop reasonably uniform and dense genetic linkage maps in pearl millet. Such genetic maps can be used in association or linkage studies to identify QTL, and occasionally SNPs [26], controlling traits of interest.

The objectives of this study were (1) to construct a genome-wide linkage map based on GBS-derived SNP markers in a pearl millet F_2_ mapping population, and (2) to map one or more major *Rf* loci governing male-fertility restoration and male-sterility maintenance in the A_4_ CMS system of pearl millet.

## Results

### Phenotypic variation in the mapping population

**Fig. 1:**
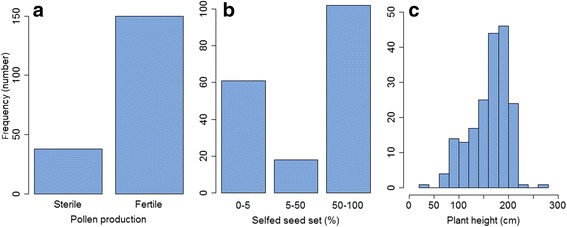
The distribution of phenotype scores for (**a**) pollen production, (**b**) selfed seed set and (**c**) plant height

All F_1_ hybrid individuals produced from the ICMA 02777 × ICMR 08888 cross were fully male-fertile, as were the selfed progeny of ICMB 02777 and ICMR 08888. The parental plant ICMA 02777 used in the cross was fully male-sterile, as were the progeny when it was crossed to its maintainer, ICMB 02777. The observation that all F_1_ plants were fully male fertile suggests dominant inheritance of male-fertility restoration in the pearl millet A_4_ CMS system. A total of 138 plants in the F_2_ population produced pollen (and hence were male fertile) and 50 plants did not produce pollen (male sterile) (Fig. 1), which fits well the 3:1 segregation ratio of a single dominant gene (χ^2^ = 0.26, *p* = 0.614). The distribution of phenotypes for selfed seed set percentage also revealed two major classes (no seed set and medium to good seed set) plus an additional low-frequency intermediate class with low to medium seed set (Fig. 1). Plant height was almost normally distributed and exhibited high variation in the F_2_ population, ranging from 38 cm to 270 cm, with an average plant height of 163 cm. This high variation for plant height, and its slightly bi-modal distribution, suggested that the F_2_ population was segregating for a recessive dwarfing gene, as well as many loci of small effect governing this trait.

### Genetic map construction based on polymorphic markers

A total of 449.5 million reads were generated by sequencing the 196 samples; 2 samples were subsequently excluded due to low sequencing quality. The 194 high-quality samples had on average 2.31 million reads (range 0.33–6.81 million) per sample. The two samples of the parental line ICMB 02777 had a total of 4,990,691 reads and the four samples of ICMR0888 had a total of 14,680,021 reads.

**Fig. 2:**
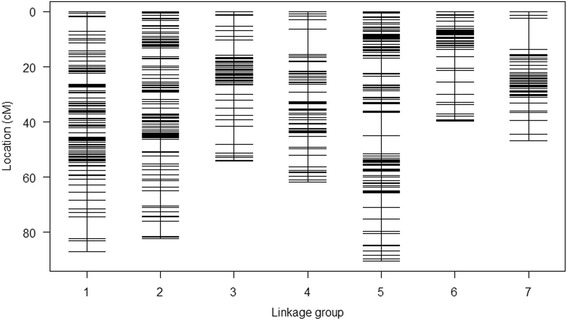
F_2_ genetic linkage map of pearl millet obtained using genotyping-by-sequencing (GBS) single-nucleotide polymorphism (SNP) markers. Each vertical bar represents one linkage group with black horizontal lines showing the SNP locations on each linkage group

A total of 160,000 raw SNPs were called using the pearl millet reference genome version 1.1 sequence [27] (kindly provided by the Pearl Millet Genome Sequencing Consortium). Filtering for high quality polymorphic SNPs reduced the number of SNPs to 2416, which were used in the first step of the map construction. The MSTmap algorithm grouped all SNPs in 7 linkage groups (LGs), except 73 outlying SNPs, which were excluded. The grouping of LGs agreed with the grouping of the reference genome sequence. The 2343 SNPs included many redundant markers which were filtered out. The final genetic map was based on 460 SNPs and had an overall length of 462.2 cM. Markers are evenly distributed (Fig. 2, Additional file 1: Table S1), with an average inter-marker spacing of 1.0 cM and a maximum spacing of 11.1 cM (Table 1). The length of the LGs ranged from 39.7 cM (LG 4) to 90.4 cM (LG 5). While this manuscript was under review, the final pearl millet reference genome was published [27] (The physical location and genetic context of all SNPs in this map are included in Additional file 2).

**Table 1:** Statistics of the pearl millet linkage map

Linkage Group	Markers	Length (cM)	Average spacing	Maximal spacing
1	93	87	0.9	7.8
2	95	82.3	0.9	5.5
3	47	54.2	1.2	6.6
4	54	61.7	1.2	9.3
5	91	90.4	1.0	8.2
6	39	39.7	1.0	4.4
7	41	46.9	1.2	11.1
overall	460	462.2	1.0	11.1

### Identification of male-fertility restoration and plant height QTLs

The SNP-based genetic linkage map was used in multiple regression analyses to identify QTL for the male-fertility restoration (determined by both pollen production and selfed seed set) and plant height. One marker interval on LG 2 was significantly associated with both pollen production and selfed seed set. For pollen production, the QTL explained 14.5% of the observed phenotypic variance, while it explained only 9.9% of the observed phenotypic variance for selfed seed set (Table 2). For plant height, one QTL was identified on LG 4, which explained 24.5% of the observed phenotypic variance.

**Table 2:** Quantitative trait loci for pollen production and selfed seed set (traits indicating male-fertility restoration of the A_4_ CMS system) and plant height

	LG^b^	Position (cM)	Support Interval (cM)	LOD	Left SNP^c^	Right SNP^c^	R^2^_adj_	Genetic Effect		Cross-Validation Frequency (%)
								Additive	Dominant	
Pollen Production^a^	2	44	42–45	7.86	S2_110825781	S2_195649011	14.48	0.381	0.437	97
Selfed Seed Set	2	44	42–45	5.39	S2_110825781	S2_195649011	9.98	0.349	0.382	38
Plant Height	4	38	37–40	11.52	S4_48328719	S4_70429741	24.54	26.686	27.697	69

^a^Pollen Production was scored as 0 = no pollen production, 1 = pollen production; Selfed Seed Set was scored as 1 = up to 5% seed set, 2 = 5 to 50% seed set, 3 = more than 50% seed set when plants were self-pollinated; Plant Height was measured in cm

^b^LG, Linkage group; 1-LOD Support Interval; LOD, logarithm of odds; R^2^_adj_, adjusted percentage of observed phenotypic variance; Frequency, QTL frequency determined by cross-validation

^c^The genomic context of all SNPs in the linkage map, along with their physical location in the current pearl millet reference genome (NCBI Assembly [27]), are presented in Additional file 2

The QTL frequency analysis showed that the QTL position for pollen production was found in 97% of the cross-validation runs, and the QTL detected for selfed seed set in 38% of the runs. The QTL for plant height on LG 4 was found in 69% of the runs. We verified the QTL analysis with a single marker regression model implemented in R/qtl to confirm the multiple regression model used within PLABMQTL. Both algorithms identified the QTL at the same positions, and had very similar proportions of phenotypic variances explained.

### Conversion of flanking SNPs to KASP assays

In order to make the two flanking SNP markers of the QTL usable for applied marker-assisted selection, they were converted into single marker assays. To enable a cheap, fast and high-throughput screening, we chose to convert them into allele-specific PCR based (KASP) markers. For both SNPs (S2_110825781 and S2_195649011) the KASP assay was successful and showed three genotypic classes (Fig. 4A). There were two haplotypes that showed a very high frequency for fertile individuals, while one haplotype had approximately equal frequency of sterile and fertile individuals (Fig. 4B, Additional file 3: Table S2). We genotyped all members of the F_2_ population and verified the functionality of the detected QTL and obtained a very similar R^2^ as observed with the original genotype data.

## Discussion

### Comparison to existing genetic linkage maps based on GBS and other markers

High-throughput sequencing technologies and the development of user-friendly software packages for sequencing analysis have advanced the options for marker detection tremendously. SNP-based linkage maps are already used in many crops, especially in those where the reference genome sequence is available. In pearl millet, two GBS-based linkage maps have been recently published. Moumouni et al. [24] published a map based on a small F_2_ population, without using a reference sequence (using the UNEAK pipeline [28] in TASSEL), while Punnuri et al. [25] used a mapping population based on recombinant inbred lines (RIL) with the same draft reference genome sequence that we used in our study (The final pearl millet reference genome was published [27] while this manuscript was in review.). The total map lengths of Moumouni et al. [24] and Punnuri et al. [25] were 717 cM and 641 cM, respectively, both substantially longer than our map (462 cM). Sehgal et al. [29] published a consensus function map based on gene-based SNPs, CISPs and EST-SSRs which was 815.3 cM. However, there are also previous genetic maps with similar or shorter total map lengths compared to ours: Qi et al. [30] published an 473 cM long map based on a F_2_ pearl millet population and 242 SSR and RFLP markers, and the original pearl millet map of Liu et al. [31] spanned only 303 cM. The total length of a linkage map is influenced by several factors including the recombination rate of the mapping population and the relatedness of the parents. Thus, precise comparison of map lengths from different studies is not meaningful so long map lengths are within the same approximate range, as is the case for our map.

Both our analysis and Punnuri et al. [25] numbered the LGs according to the concensus map of Rajaram et al. [20]. However, the relative lengths of the LGs were quite different in our map and those reported by Punnuri et al. [25]. Especially LG 3 and LG 6, which were relatively short in our map (54.2 cM and 39.7 cM, respectively), were quite long in the map of Punnuri et al. [25] (175 cM and 112 cM). Based on base pairs given in the pearl millet genome sequence, LG3 is the longest of the seven LGs and LG 6 is the 4th in length. One probable reason for the difference in length may lay in the used restriction enzyme chosen for GBS. We used the enzyme *Pst*l while Punnuri et al. [25] use *Ape*KI The relative lengths of the LGs in the consensus map of Rajaram et al. [20] were much closer to the LGs relative lengths of our map than to those on the map of Punnuri et al. [25].

The two existing GBS-based linkage maps have all higher marker densities than previous maps based on other marker types. The map of Punnuri et al. [25] showed a higher density than our map, which in turn is denser than the map of Moumouni et al. [24]. The higher density reported by Punnuri et al. [25] was expected because they used a RIL mapping population, which has a higher recombination rate (effectively double that of an F_2_ population of similar size and parentage). However, we can still classify our map as mostly dense, uniformly- and well-saturated, because there was only one gap with more than 10 cM between adjacent markers. The integrated EST-SSR + DArT marker-based pearl millet linkage map reported by Ambawat et al. [21] spanned 740 cM (Haldane), with an average adjacent-marker distance of 2.7 cM. This map had been constructed using a RIL population of 140 individuals from a cross of inbred lines that are expected to segregate for not only the *d2* dwarfing gene, but also for male-fertility restoration and male-sterility maintenance for both the A_1_ and A_4_ CMS systems of pearl millet. Testcrossing that RIL population to iso-nuclear seed parents 81A_1_ and 81A_4_ would permit independent confirmation of our results for A_4_, as well as demonstrating the relationship, if any, between fertility restoration / sterility maintenance loci for these two commercially exploited pearl millet CMS systems. The superior genomic coverage of the map of Ambawat et al. [21] in peri-telomeric regions of most linkage groups could also help identify modifiers of any major fertility restoration / sterility maintenance loci detected for either of these two CMS systems. The utility of genic markers detected using the *Pst*I endonuclease for ensuring marker coverage in such regions was demonstrated by Ambawat et al. [21] when they were able to map a major gene for rust resistance that had previously proven “un-mappable” as its position was more distal than any RFLP or SSR marker at the top of LG 1.

### Inheritance of male-fertility restoration

In crops where seed or fruit comprise the economic harvest, the restoration of male fertility in F_1_ hybrids is usually an important prerequisite for an economically viable hybrid cultivar that are harvested prior to flowering (such as beets, carrots, leeks and onions), are examples of crops in which hybrid cultivars need not have restored male fertility as are parthenocarpic cucumbers. Similarly, many forages and most ornamentals need not have restored male fertility because seed set is not required for their use in agriculture.

Gupta et al. [16] showed that male-fertility restoration in the A_4_ CMS system of pearl millet followed a monogenic dominant pattern of inheritance using phenotyping procedures similar to those we have used. Our observations are in line with this result because we also found a 3:1 (male-fertile:male-sterile) segregation pattern in the F_2_ population. However, the assumption of single gene-control based on the phenotypic data does not seem certain yet because the results of our mapping study indicated that there could also be minor genes.

Previous studies on the A_4_ CMS system in pearl millet have demonstrated its stable male sterility and reliable male fertility restoration across Indian environments. A number of seed parent pairs (male-sterile A-lines and their iso-nuclear B-line maintainers) based on the A_4_ CMS system are now available to pearl millet breeders in South Asia and sub-Saharan Africa, as well as in the Americas. Our phenotypic data suggest that a substantial portion of this stability may be due to simple inheritance of male-sterility maintenance and male-fertility restoration in this system (compared to 1-, 2- and 3-gene male-fertility restoration found for A_1_; CT Hash unpublished). In this case, one can reasonably expect similar stability for both sterility and restoration in West African environments.

### Detection of male-fertility restoration and plant height loci

**Fig. 3:**
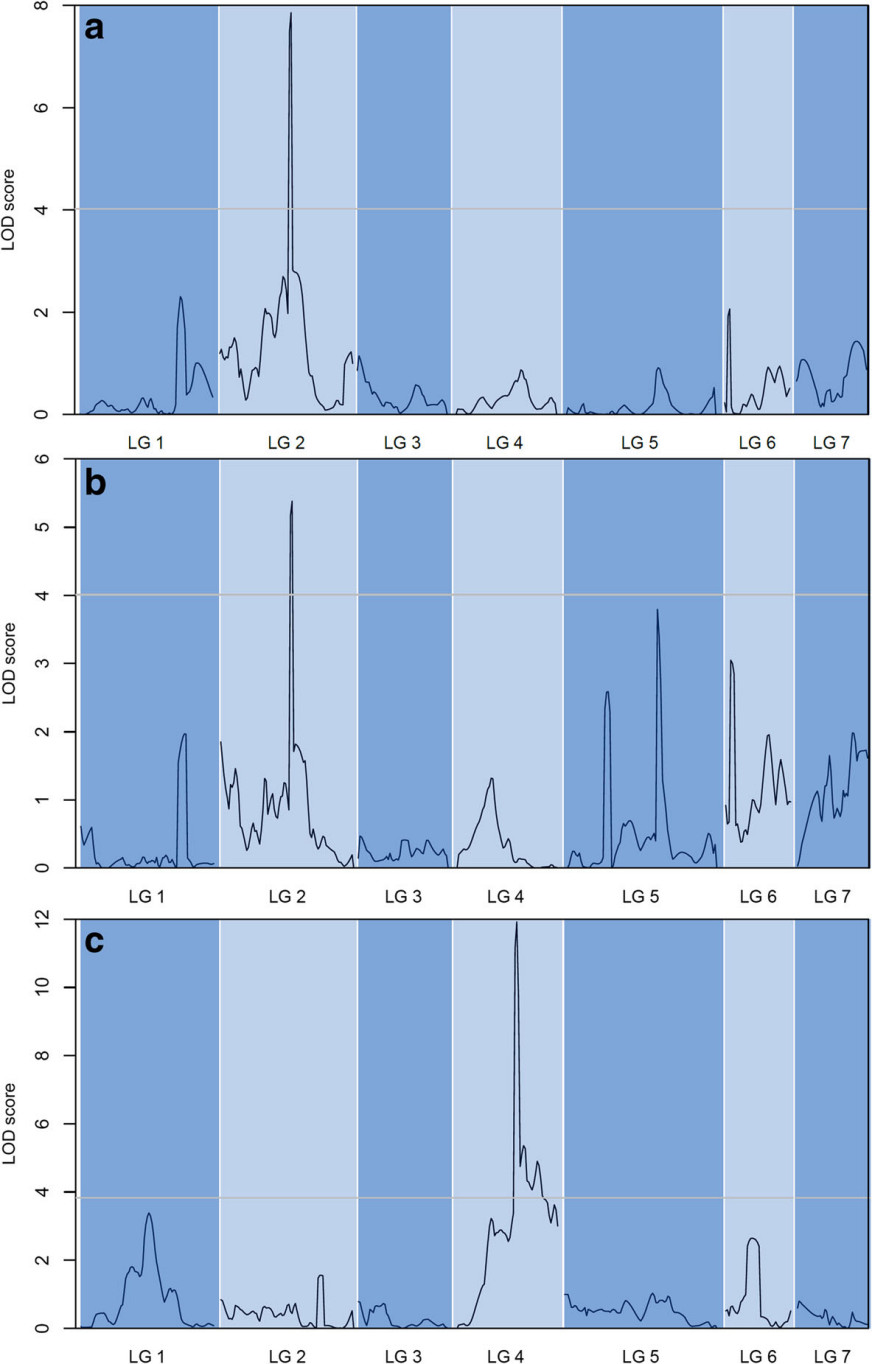
LOD curves from QTL mapping; male-fertility restoration / male-sterility maintenance for pearl millet’s A_4_ CMS system using F_2_ pollen production (**a**) and selfed seed set (**b**), and for plant height (**c**)

The QTL analysis of this study identified a major fertility restoration / sterility maintenance locus of the A_4_ CMS system on LG 2. Assuming single-gene control, we expected that the identified locus would explain a relatively high percentage of observed phenotypic variation. However, the estimated R^2^_adj_ values were only 14.5% and 9.9% for pollen production and selfed seed set, respectively, which were significantly below our expectations. This discrepancy might be affected by some minor or modifying *R*_*f*_ genes that could not be detected in this QTL analysis. The assumption of modifying genes is supported both by the observed frequency distribution (Fig. 1b) and by the LOD score curve for selfed seed set (Fig. 3b), with the latter showing some peaks that are just below the LOD threshold (e.g. on LG 1, LG 5 and LG 6). Such non-significant loci might be associated with minor *R*_*f*_ genes, although especially those detected on LG 5 are more likely to be associated with protogynous period or stigma receptivity given that they were detected only for selfed seed set and not for pollen production.

Cross-validation strengthened the evidence for high accuracy of this major *R*_*f*_ gene position, as cross-validation runs identified this same QTL for pollen production and selfed seed set score 97% and 38% of the time, respectively. The lower R^2^_adj_ and QTL frequency of selfed seed set compared to pollen production might be caused by those plants with low to intermediate (5–50%) seed set. Low seed set in fertile plants can be caused by several factors, such as partial male-fertility, a combination of short stigma receptivity with long protogynous period (time between first stigma emergence and initiation of anthesis on the same panicle), heat stress (as our screening was done in the hot season) and/or insect-feeding damage to stigmas. Male-sterile plants can show higher-than-expected seed set due to pollen contamination inside the selfing bag due to poor closure of the base of the bag, bag entry by pollen-bearing insects, or by the glued corners of the selfing bag opening during sprinkler irrigation or rainfall. In contrast, classification of anthers as sterile and fertile was more distinct, thus, we can assume a smaller error rate for pollen production as compared to selfed seed set.

**Fig. 4:**
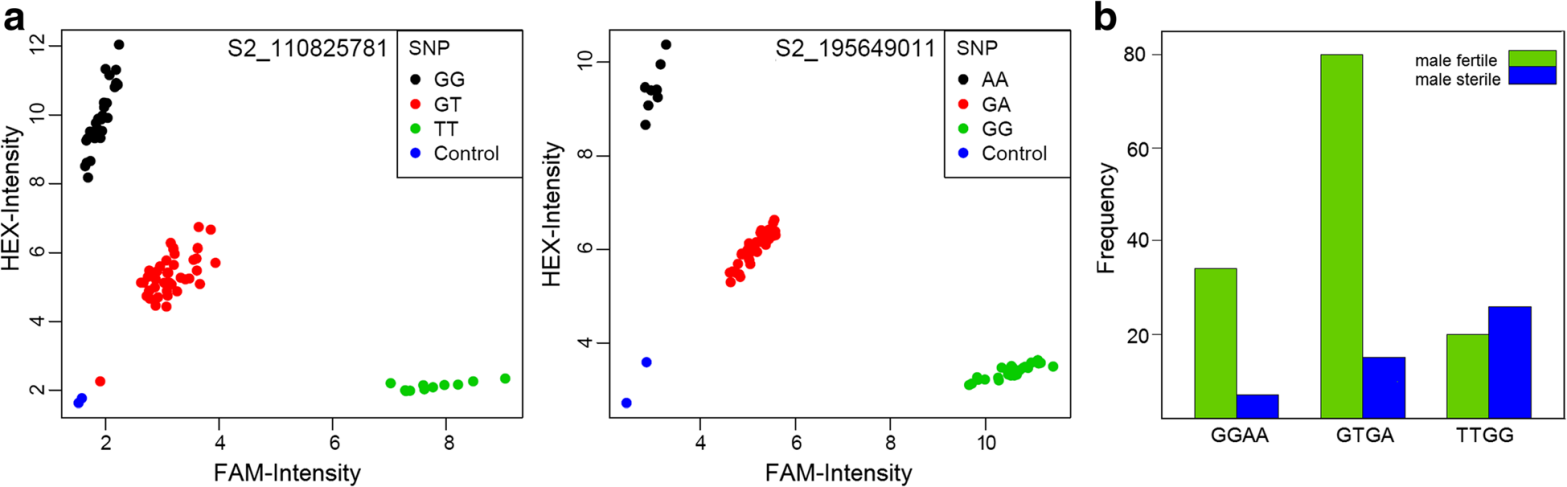
**a** Development of functional markers for the SNPs S2_110825781 and S2_195649011 and (**b**) Bar plot of male fertile and male sterile plants of the three haplotypes from these two markers

Based on the developed KASP markers for the QTL detected by pollen production, we saw that fertile individuals could be predicted with reasonable accuracy, while sterile genotypes would not be well predicted (Fig. 4b). This indicates that at this stage our KASP markers would be appropriate to select for restorer types, but not for maintainers. This finding is certainly linked with the relative low R^2^ value, and should be validated in future studies, to develop KASP markers that are also suitable to select maintainer lines.

Plant height was analyzed as a reference trait because our mapping population segregated for a dwarfing gene (*d*_2_); this gene was previously mapped to LG 4 by Azhaguvel et al. [32] and Parvathaneni et al. [33]. Since we also identified one major height QTL on LG 4 (R^2^_adj_ = 24.5%), we can assume that this locus is associated with *d*_2._ Finding this QTL on the same linkage group was used as a cross-check for the correctness of our other QTL analyses. However, Azhaguvel et al. [32] estimated that the *d*_2_ locus on LG4 explained 64% of observed phenotypic variance, which is much higher than the R^2^adj. value we estimated (24.54%). The most likely reason for this is that the population used by Azhaguvel et al. [32] was derived from a cross of two non-allelic semi-dwarf lines, and so had a substantially smaller proportion of tall plants than did our F_2_ population. They found additionally on LG 1 the locus of the *d*_*1*_ dwarfing gene. In our LOD curve for plant height there is one peak on LG1 (Fig. 3c) which is just below the LOD threshold, that is presumably associated with the *d*_*1*_ locus originally mapped by Azhaguvel et al. [32].

### Importance for future breeding programs

Our study and that of Punnuri et al. [25] have shown that GBS-SNP-based linkage maps, based on F_2_ or RIL mapping populations, are suitable for QTL detection in pearl millet due to high marker saturation. GBS is currently the most informative and cost-effective marker type, but it should be noted that the high marker number achieved by GBS cannot entirely be exploited in an F_2_ mapping population due to its lower recombination rate (and therefore higher marker redundancy) compared to RILs. Validation of our results using a RIL population is required in order to verify the existence of further minor genes modifying the fertility restoration in the A4 cytoplasm.

This study identified a major male-fertility restoration / male-sterility maintenance gene for the A_4_ CMS system of pearl millet*,* which is a crucial step in understanding the genetic basis of this economically important trait. Knowledge of the gene location will offer pearl millet breeders more efficient strategies to develop male parents carrying the major *Rf* allele. Introgression of the restoration allele by integrated conventional and marker-assisted selection will save time, compared to introgression based on purely phenotypic selection. The resources saved by using an integrated approach can then be used to develop a higher number of strongly-restoring hybrid male parents for the A_4_ CMS system or allocated to other parts of the breeding program. Especially in West Africa, where hybrid breeding is just starting and where restorer genotypes are relatively uncommon in local landrace and improved open-pollinated genotypes [17], more efficient introgression of restorer genes will be highly beneficial.

Similarly, this study is a first step towards efficient introgression of the major A_4_ maintainer allele (*rf*) into seed parent genepools. Efficient introgression will allow heterotic pools in pearl millet to be built up independently of the maintainer/restorer characteristics of specific germplasm, thus allowing breeders to focus on genetic diversity, combining ability, and agro-morphological traits.

## Conclusions

The phenotypic data of this study and that by Gupta et al. [16] indicate a monogenic inheritance of the A_4_ male-fertility restoration / male-sterility maintenance. Such inheritance is desired in hybrid breeding, as it is relatively simple to introgress and is usually little influenced by the environment. However the unexpected low variance explained by our mapped QTL suggests the presence of minor or modifying genes. Future studies using RIL populations, should investigate whether the fertility restoration of the A_4_ system is influenced by only one major gene, by several additional minor genes, or by more than one major gene depending upon the genetic backgrounds of the parents. This could explain the relatively low portion of the observed phenotypic variance explained by the QTL in the present study. Beside this verification, the developed KASP markers can be used for high-throughput screening of the desired haploblock in applied pearl millet hybrid breeding, thereby facilitating development of pearl millet hybrid parents.

## Methods

### Plant material

An F_2_ mapping population of 190 plants was developed for this study. Plants were segregating for A_4_ male-fertility restoration as the primary target trait and *d*_2_ dwarf plant height as the secondary target trait. This F_2_ mapping population was produced at the ICRISAT Sahelian Center, in Niamey, Niger, by selfing F_1_ plants derived from a single plant × plant cross of inbred lines ICMA 02777 × ICMR 08888. The 190 plants were generated by advancing three sub-populations of 70 F_2_ plants each, the sub-population being derived by selfing a single F_1_ plant. A portion of sown seeds did not establish plants and could therefore not be phenotyped. The A_4_-cytoplasm male-sterile line ICMA 02777 was derived from ICMB 02777 by backcrossing its nuclear genome to 81A_4_ cytoplasm source and is homozygous for semi-dwarf plant height at the *d*_2_ dwarfing gene locus. The pedigree of ICMB 02777 is HHVBC-II HS-9-1-1-2-7-1, in which HHVBC-II is the second High Head Volume B-Composite bred at ICRISAT-Patancheru, and has a substantial portion of its genetic background derived from *Iniadi* landrace germplasm from Togo. The restorer line ICMR 08888 was bred at ICRISAT-Patancheru by selfing within improved synthetic variety ICMS 7704, which is genetically tall at the *d*_2_ locus. ICMR 08888 has the pedigree ICMS 7704-S1–52–3-1-2-1-2-1-6-B-B, indicating that this inbred is derived from the 52nd S1 progeny of ICMS 7704 that was evaluated, and that seven generations of single-plant selection with selfing were followed by two generations of advance of bulks of seed from two or more selfed plants. Seed parent pair ICMA 02777/ICMB 02777 and restorer line ICMR 08888 were both developed at ICRISAT-Patancheru. Although they are relatively long-duration for Indian dryland conditions, their lifecycles are generally too short for most pearl millet producing regions in West and Central Africa.

### Phenotyping

The F_2_ population of 190 plants plus their parental lines were raised under irrigated conditions at the ICRISAT research station in Sadoré, Niger during the dry season, planted to the 16th of March, 2014. The crop was grown as single plants per hill under irrigation with recommended fertilization. At the five-leaf stage a single leaf was collected from each plant into a labelled coffee filter, with the stapled and labelled coffee filters placed in zip-lock plastic bags containing silica gel desiccant, and the plastic bags then stored with additional desiccant in an air-conditioned seed store until they could be shipped for DNA isolation and genotyping. At the boot-leaf stage, two emerging panicles per plant were covered with semi-transparent parchment paper bags closed with a paper clip to enforce self-pollination, with later-appearing panicles being left uncovered to facilitate observation of anther structure, pollen shed, and open-pollinated seed set. During the pollen shedding period, anthers of plants were classified as male-fertile (bearing pollen-producing anthers) or male-sterile (bearing only shrunken anthers with no pollen). Hereafter, this trait will be referred to as pollen production. It could be scored on 188 F_2_ plants.

At maturity, two panicles of each F_2_ plant were scored for selfed seed set as an additional phenotype to assess the target trait male-fertility restoration; the scoring system was: 1 = up to 5% selfed seed set from the total number of flower buds (male sterile), 2 = 5 to 50% selfed seed set (partially male -fertile); 3 = more than 50% selfed seed set (male fertile). Selfed seed set could be scored on 181 plants because of selfing bag losses from some plants due to strong winds.

The F_2_ population segregated for the *d*_*2*_ dwarfing gene, which has already been mapped in previous studies [32, 33] and was intended as a reference trait to verify the quality of our linkage map and F_2_ population. We recorded plant height (cm) on all 190 F_2_ plants.

For pollen production we tested a 3:1 segregation ratio of male-fertile:male-sterile plants using a χ^2^-test.

### DNA extraction, genotyping-by-sequencing and SNP calling

Genomic DNA was extracted from dried young leafs of individual F_2_ plants and their parental lines using the DNeasy Plant Mini Kit (Qiagen Inc., Valencia, CA). Quality and quantity check of extracted DNA was performed using *Hind*III digestion and gel analysis. Fifty μl aliquots of each of 196 DNA samples (190 F_2_ individuals and six parents) containing > 10 ng μL^− 1^ per sample were sent in three 96-deep well plates to the Genomic Diversity Facility at Cornell University in Ithaca, New York, for GBS analysis. The remaining space in the plates was filled with further pearl millet samples from our project. Each 96-well plate contained one randomly positioned blank.

GBS libraries were prepared and analyzed at the Genomic Diversity Facility at Cornell University according to Elshire et al. [22], using the restriction enzyme *Pst*I and sequenced at 96-plex level on the Illumina HiSeq2000 with single-end read sequencing.

The raw GBS data files (FASTQ) were processed to SNP calls using the GBS version 2 pipeline of Tassel 5 (Version 5.2.28) [34]. The sequenced tags were aligned to the pearl millet reference genomic sequence provided by the Pearl Millet Genome Sequencing Consortium [27], using the Burrows-Wheeler Alignment Tool (BWA) [35].

### Quality check and genetic map construction

High-quality SNPs were called using TASSEL 5. SNPs with more than 20% missing data, a minor allele frequency below 40%, or those which were heterozygous in one or both parents were filtered out. Genotypes (plants) showing > 50% missing data were removed. After this filtering, the remaining 2445 SNPs were imputed using the FSFHap algorithm [36] implemented in TASSEL 5.

Chi-square tests were performed on each marker for 1:2:1 (A:H:B) expected genotypic segregation ratios to assess the amount of segregation distortion. Only 29 SNPs showed significant segregation distortion at the 5% level after a Bonferroni correction for multiple tests. These SNPs were discarded.

The genetic map was constructed using the MSTmap algorithm [37] implemented in the R package ASMap [38, 39]. A total of 73 SNP markers were designated to outlying linkage groups (LG) with a very low number of SNPs and were discarded. The numbering of LGs was based on the genome sequence, which corresponds to the numbering of the consensus map published by Rajaram et al. [20]. The map length was re-estimated using the Lander-Green algorithm within the software package R/qtl, and choosing the Haldane function. The genetic map with its 2343 markers contained many redundant markers (caused by co-segregation) which were excluded, thus the final linkage map was based on 460 markers.

### QTL mapping

QTL analysis was performed with the software PLABMQTL [40] using composite interval mapping based on multiple regression [41]. The QTL mapping model included additive and dominance effects, and cofactors were chosen by stepwise regression.

The critical logarithm of odds (LOD) scores were determined empirically according to Churchill and Doerge [42] using 1000 permutation runs and α = 0.05. The LOD thresholds were for pollen production = 4.02, for selfed seed set = 4.01, and for plant height = 3.83. The adjusted proportion of the phenotypic variance explained by the individual QTL (R^2^_adj_) was calculated. To assess the quality of results of QTL detection, the occurrence of the QTL (QTL frequency) within a 1-LOD support interval was determined by conducting 1000 five-fold cross-validation runs [40]. Due to the non-normal distribution of phenotypes, we also fitted a logistic regression to the data using the glm() function in R. The results were almost identical to the original results, thus they were not considered further.

### KASP-marker development

The two flanking SNPs of the major QTL on LG 2 were converted into KASP assays. SNP S2_11085781 was converted into KASP assay PM_S2_11085781, which comprised the two allele specific primers PM_S2_11085781_T (5’-FAM-TailSeqGGAACCATCGCAACATCGTAAGA-3′) and PM_S2_11085781_G (5’-HEX-TailSeq-GGAACCATCGCAACATCGTAAGC-3′) and the common primer PM_S2_11085781_Com (5’-GGGTTGAAGACCAGAGGATAGTCTGC-3′). SNP S2_19564901 was converted into KASP assay PM_S2_19564901, which comprised the two allele specific primers PM_S2_19564901_G (5′- FAM-TailSeqCTCGTTGGTCAGAATGGACATCAG-3′) and PM_S2_19564901_A (5’-HEX-TailSeq-CTCGTTGGTCAGAATGGACATCAA-3′) and the common primer PM_S2_19564901_Com (5′- ACGCAACATTCCCTAAGCGAAGTT-3′). For both KASP assays the FAM-allele corresponds to the sterile parent and the HEX allele to the fertile parent. Both assays were run as 6 μl PCR reactions, with a standard KASP 61–55 °C touchdown PCR program (http://www.lgcgroup.com/products/kasp-genotyping-chemistry/kasp-technical-resources/) on a Roche LightCycler®480II instrument.

## Additional files

Additional file 1: **Table S1.** Distances between SNP marker in the genetic linkage map. (XLSX 24 kb)

Additional file 2: SNP marker information including the location in the reference genome. (TSV 1238 kb)

Additional file 3: **Table S2.** List of all F2 individuals, their haplotypes based on the two functional markers developed for the the identified QTL, and their phenotypes. (XLSX 17 kb)

## Abbreviations

CMS: Cytoplasmic male sterility
GBS: Genotyping-by-sequencing
LG: Linkage Group
LOD: Logarithm of odds
QTL: Quantitative trait locus
SNP: Single nucleotide polymorphism
